# Automated interpretation of fundus fluorescein angiography with multi-retinal vascular lesion segmentation

**DOI:** 10.3389/fmed.2026.1762735

**Published:** 2026-04-07

**Authors:** Ziwei Zhao, Shoujin Huang, Fan Song, Weiyi Zhang, Yan Lu, Xianwen Shang, Yingfeng Zheng, Danli Shi, Mingguang He

**Affiliations:** 1 School of Optometry, The Hong Kong Polytechnic University, Hong Kong, Hong Kong SAR, China; 2 Research Center for SHARP Vision, The Hong Kong Polytechnic University, Hong Kong, Hong Kong SAR, China; 3 Shenzhen Technology University, Shenzhen, China; 4 Foshan Second People's Hospital, Foshan, China; 5 State Key Laboratory of Ophthalmology, Guangdong Provincial Key Laboratory of Ophthalmology and Visual Science, Zhongshan Ophthalmic Center, Guangdong Provincial Clinical Research Center for Ocular Diseases, Sun Yat-sen University, Guangzhou, China; 6 Center for Eye and Vision Research, Hong Kong, Hong Kong SAR, China

**Keywords:** convolutional neural networks, deep learning, lesion segmentation, retinal fluorescein angiography, retinal vascular disease

## Abstract

**Purpose:**

Fundus fluorescein angiography (FFA) is essential for diagnosing and managing retinal vascular diseases, yet its evaluation is time-consuming and subject to inter-observer variability. We aim to develop a deep-learning-based framework for multi-lesion segmentation in FFA images and to evaluate its primary performance on standard 55 ° images.

**Methods:**

A dataset of 428 standard 55 ° FFA images and 53 ultra-wide-field (UWF) FFA images was annotated for non-perfusion areas (NPA), microaneurysms (MA), neovascularization (NV) and laser spots. A residual U-Net framework was trained using a data-pooling strategy. The primary analysis was performed on the 55 ° test set, whereas the UWF subset was analyzed only as a preliminary exploratory observation because of its limited sample size. Performance was assessed using Dice score, Intersection over Union (IoU) and recall.

**Results:**

On 55 ° FFA images, the model achieved Dice scores of 0.65 ± 0.24 for NPA, 0.70 ± 0.13 for MA, 0.73 ± 0.23 for NV, and 0.70 ± 0.17 for laser spots. In an exploratory analysis of UWF images, overlap-based performance was lower for NPA (0.48 ± 0.21, *p* = 0.02) and MA (0.58 ± 0.19, *p* = 0.01), whereas laser spot segmentation was similar. NV segmentation in CNV achieved a Dice score of 0.90 ± 0.09. NPA segmentation was better in RVO than in DR, whereas MA segmentation was better in DR than in RVO. Moreover, NV segmentation was significantly stronger in the venous phase (0.77 ± 0.17) and late phase (0.75 ± 0.24) than in the arteriovenous phase (0.50 ± 0.32, both *p* < 0.05). Exploratory UWF analysis showed lower overlap-based performance, highlighting the likely impact of domain shift and the need for larger UWF-specific datasets.

**Conclusion:**

This study has established a highly consistent and reproducible framework for multi-lesion segmentation in FFA images. The model may help standardize lesion quantification, reduce manual grading burden, and support future development of larger multi-center FFA datasets.

## Introduction

Retinal vascular diseases, including diabetic retinopathy (DR), retinal vein occlusion (RVO) with its subtype branch retinal vein occlusion (BRVO), and neovascular age-related macular degeneration (nAMD) coupled with choroidal neovascularization (CNV), pose a substantial global health challenge. As leading causes of vision loss and blindness, these diseases affect millions and impose considerable personal and socioeconomic costs (1). Specifically, DR prevalence in diabetics is 27.0%, contributing to 0.4 million cases of blindness (2), and the projected incidence of AMD by 2040 is 288 million, with about 10% expected to develop neovascular complications (3).

Fundus fluorescein angiography (FFA) is a critical diagnostic tool to detect and characterize retinal vascular abnormalities such as microaneurysms (MA), neovascularization (NV), and capillary non-perfusion areas (NPA). Segmenting and quantifying these lesions are critical for accurate diagnosis, disease staging, and monitoring. For instance, in DR, the extent of NPA indicates the severity of ischemia and predicts the risk of disease progression, thereby determining the necessity for laser photocoagulation (4). Similarly, measuring MAs provides valuable prognostic information (5). In DR, MA turnover rates serve as key indicators for disease progression (6–8). While historically overlooked in RVO, recent studies link MA to persistent macular edema (ME) in BRVO (9, 10). Notably, MAs in collateral vessels independently predict retinal edema (11). Thus, precise identification of these biomarkers helps identify patients at high risk of vision-threatening complications (12).

However, accurate interpretation of FFA images is inherently complex and subject to inter-observer variability (13). While precise quantification of lesions (e.g., measuring the exact area of non-perfusion) holds critical prognostic value for risk stratification, it is rarely performed in daily practice because manual delineation is prohibitively time-consuming (14). An automated segmentation system addresses these challenges by enabling highly consistent and reproducible quantification without adding to the clinical burden, thereby streamlining the workflow for busy clinicians (15). Furthermore, it may assist less experienced ophthalmologists by highlighting subtle or scattered lesions and reducing the risk of missed findings.

Recent work has explored deep learning (DL) for DR grading and lesion detection (16). Pan et al.'s study achieved multi-label detection in DR using FFA, with sensitivities of 79.7% for NPA, 98.0% for MA, 84.0% for leakage, and 80.3% for laser scars (17). However, their focus has predominantly been on image-level lesion classification without specific localization or delineation. Deep learning approaches, including convolutional neural networks (CNNs), have shown promise in segmenting FFA lesions, as summarized in Table 1. These studies DL's potential or the potential of deep learning for accurate lesion identification in FFA images of specific disease datasets (15, 18–25). Nevertheless, limitations exist: research has been focused only on 30 °, 55 ° or ultra-widefield (UWF) FFA images, targeting specific lesions within particular retinal diseases such as NPA in DR (18, 19, 24), NPA in BRVO (20, 22), and CNV in nAMD (25). This narrows their application and prevents the identification of multiple lesions across a range of diseases. Meanwhile, as DL models advance in accuracy, there is an accompanying surge in the volume of data required to train, validate, and test these models.

**Table 1:** Summary of previous studies on lesion segmentation performance in FFA images.

Year	Author	Modality	Target	Disease	Test model	Best F1 score
2020	Joan et al.	UWF	NPA	DR	U-net	0.661
2020	Jin et al.	30 °	NPA	DME	U-net	N/A
2021	Tang et al.	55 °	NPA	RVO	CE-net, U-Net, DeepLabv3+	0.883
2021	Chen et al.	55 °	Leakage	CSC	attention gated network	0.811
2022	Kanato et al.	55 °	NPA	RVO	U-Net, PSPNet, DeepLab v3+	0.736
2022	Xiang et al.	55 °	NPA	Not Specified	ResNet50+VGG16+MLFB+SAT	0.772
2022	Lee et al.	UWF	NPA NV	DR	DeepLab v3+, SegNet, U-net	0.67 0.87
2023	David et al.	55 °	NV Leakage	nAMD	U-net based	0.73 0.65
2023	Zhao et al.	55 °	NPA	DR & BRVO	ResNet-152, Unet-VGG16	0.90

FFA, fundus fluorescein angiography; UWF, ultra-widefield imaging; DR, diabetic retinopathy; RVO, retinal vein occlusion; BRVO, branch retinal vein occlusion; CSC, central serous chorioretinopathy; nAMD, neovascular age-related macular degeneration; NPA, non-perfusion areas; NV, neovascularization.

To address these gaps, we assembled a pixel-level annotated FFA dataset containing NPA, MA, NV, and post-photocoagulation laser spots across multiple retinal vascular conditions. Rather than claiming architectural novelty, the main novelty of this study lies in its clinical task formulation: a unified framework for concurrently segmenting multiple morphologically distinct lesions across DR, RVO, and CNV. In addition, to our knowledge, this is the first study to explicitly incorporate laser-spot segmentation as a dedicated class, which is clinically relevant for estimating the true ischemic burden in previously treated eyes. Overall, we propose an automated, highly consistent and reproducible approach for concurrent lesion segmentation in FFA images.

## Materials and methods

**Figure 1:**
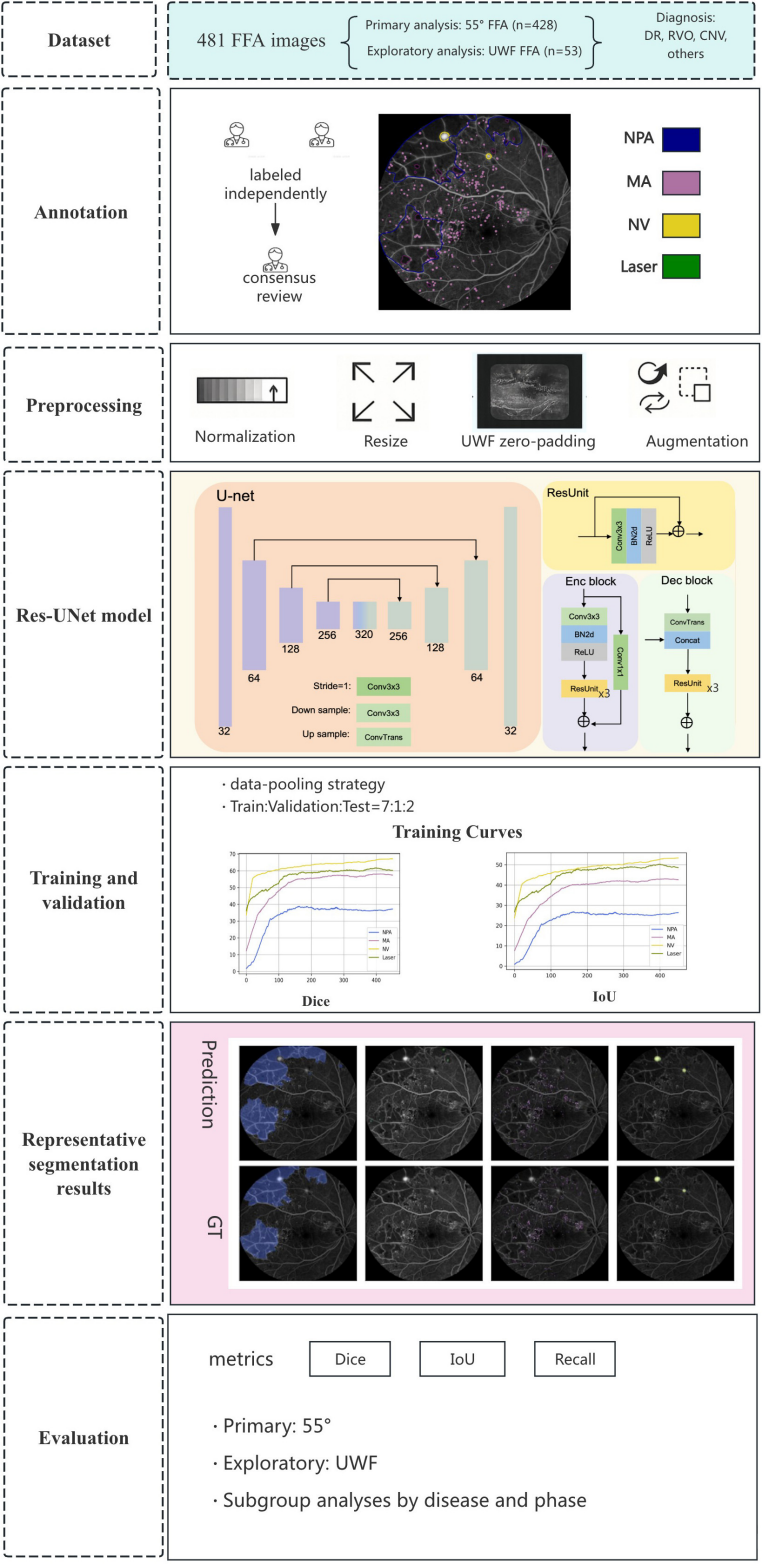
Flow diagram of this study.

The flow chart of the study is shown in Figure 1.

### Dataset

The dataset was collected from multiple tertiary hospitals and comprised 428 55 ° and 53 UWF images from 481 eyes of 481 patients. Standard 55 ° images were captured using Zeiss FF450 Plus (Carl Zeiss Meditec, North America) and Heidelberg Spectralis (Heidelberg Engineering, Heidelberg, Germany) cameras at a resolution of 768 × 768 pixels. UWF images were captured by the Optos California (Optos plc, Dunfermline, Scotland, UK) at a higher resolution of 1,536 × 1,024 pixels. Because the UWF subset was small, it was retained only as a preliminary exploratory cohort and was not used to support formal claims of cross-modality generalizability. The dataset included DR, CRVO, BRVO, nAMD, CNV, normal eye, and other diseases, and covered arteriovenous, venous, and late phases. Images with moderate obscuration, including regional masking by hemorrhage, were retained to better reflect real-world clinical practice. Only images of extremely poor quality—where retinal structures were nearly indiscernible and lacked clinical reference value—were excluded. All patient data were anonymized and de-identified following the Declaration of Helsinki. Individual consent was waived due to the retrospective nature and the thorough anonymization process. The study was approved by the Institutional Review Board of the Hong Kong Polytechnic University (HSEARS20240301004).

### Labeling and image preparation

Two experienced ophthalmologists (Z. Z. and F. S.) annotated the dataset using Labelme (*Ver 3.16.2*) independently. For each image, graders used the polygon tool to outline the optic disc, macular region, non-perfusion areas (NPA), neovascularization (NV), and laser spots (Laser), while the point tool was applied to identify microaneurysms (MA). They also determined the most likely diagnosis and the phase of the image. NPAs were defined as regions exceeding the physiological intercapillary distance yet devoid of fluorescein filling. For NV, the annotation target included the hyperfluorescent neovascular complex together with its associated leakage cloud when present, because progressive leakage is a defining angiographic feature of active neovascularization. The laser-spot class predominantly comprised old pan-retinal photocoagulation scars, which were grouped as a single category because they shared the same clinical meaning of previously treated, non-functioning retinal tissue.

Following Joan M. Nunez do Rio et al., non-informative peripheral areas in UWF images were excluded by defining a ‘gradable area'—a zone on UWF-FFA images where lesions and anatomical structures are discernible (18). The initial inter-grader Dice score was 0.69. Subsequently, the ground truth (GT) was established through consensus between the 2 graders and confirmed by a retinal specialist.

Before feeding each input image into the network, we performed min–max normalization to scale pixel intensities to the range of 0 to 1. UWF images were zero-padded to a square canvas before resizing to the network input size, rather than being directly warped, whereas standard 55 ° images were resized after normalization. Data augmentation methods included Gaussian modifications, brightness and contrast adjustments, resolution reduction, and gamma shifts, with assigned probabilities of 10%, 15%, 25%, and 10%, respectively. Random horizontal and vertical flips were also applied to both the images and labels. These augmentation steps were intended to improve robustness while preserving clinically relevant lesion morphology.

### Network architecture

Our network architecture was built on the U-net framework (26), incorporating residual units (27), in both the encoding and decoding stages as shown in Figure 1. In the encoder, input features were down sampled through 3 x 3 convolutions with a stride of 2 and then passed through repeated residual blocks, with shortcut summation between the block input and output. In the decoder, features from the skip connections were concatenated with incoming features before repeated residual processing, and the encoded representation was progressively restored.

Instance normalization and ReLU activation were applied after all convolutional operations. The channel numbers were 32, 64, 128, 256, and 320. Three residual units were used at each up sampling/down sampling stage. The network was configured with 1 input channel and 10 output channels. For the segmentation task, the five target classes were modeled as five binary segmentation tasks.

### Training and testing details

During training, the 55 ° and UWF images were combined using a data-pooling strategy rather than a dedicated domain-adaptation module. The primary objective of this design was to learn a unified representation from the larger 55 ° dataset while allowing limited exposure to UWF images. Given the small size of the UWF cohort, its role was exploratory and its results were interpreted cautiously. We employed the SGD optimizer with the Poly learning rate decay algorithm. The learning rate was set to 0.01, with a weight decay of 3e-5, batch size of 1, and momentum of 0.99. The U-net model was trained for 500 epochs, and the final model was selected according to the best mean Dice score on the validation set. The loss function combined Dice loss and cross-entropy loss. Loss was computed separately for each category and then summed for backpropagation. Training was performed on a Tesla V100 with an Intel(R) Xeon(R) Gold 6248R CPU (Intel Corporation, Santa Clara, CA, USA).

### Performance metrics

For image inference, we implemented a testing augmentation strategy, involving random horizontal and vertical flips applied to the input image into the network to obtain diverse outcomes, respectively. Subsequently, the predicted probability values were averaged to derive the final segmentation result.

The performance of the model was quantitatively evaluated using the Dice score and the Intersection over Union (IoU). Pixel-level sensitivity (recall) was additionally considered as a complementary detection-oriented metric, because overlap-based metrics alone may underrepresent clinical usefulness when a lesion is detected but its boundary is imperfectly delineated.

The Dice score, also known as the F1 score in binary segmentation, measures the similarity between the predicted ground-truth masks and is defined as:


$$\begin{array}{l} DSC=\frac{2\times |X\cap Y|}{\left|X|+|Y\right|} \end{array}$$ (1)

Here, *X* and *Y* represent the predicted and ground truth pixel sets, respectively. The Dice coefficient ranges from 0 (no overlap) to 1 (perfect agreement).

The IoU metric, also known as the Jaccard index, evaluates the overlap between the predicted segmentation and the ground truth masks and is defined as:


$$\begin{array}{l} IoU=\frac{\left|X\cap Y\right|}{\left|X\cup Y\right|} \end{array}$$ (2)

Similarly, *X* and *Y* denote the predicted and ground truth pixel sets. IoU values range from 0 to 1, with 1 indicating perfect overlap.

## Statistical analysis

Statistical analyses were performed using R software. Data are presented as mean ± standard deviation (SD). Group comparisons were conducted using two-tailed independent *t*-tests, with statistical significance set at *p* < 0.05.

## Results

### Dataset compilation

Our dataset comprised 428 55 ° FFA images and 53 UWF-FFA images, totaling 481 FFA images. For a focused study, we prioritized the 55 ° images due to their larger quantity, while also including UWF images for preliminary model performance evaluation on a smaller sample size. For the 55 ° FFA images, 305 were allocated for training, 38 for validation, and 85 for testing. Correspondingly, the UWF FFA images were divided into 40 for training, 3 for validation, and 10 for testing.

### Distribution of diseases

The dataset comprised diverse retinal pathologies, including 296 images of DR, 29 of CRVO, 57 of BRVO, 58 of nAMD or CNV, 16 of normal fundus, and 25 of other conditions requiring further examination.

### Label frequency

The comprehensive distribution of label frequencies and pixel counts is detailed in Table 2. Regarding lesion density, the mean number of labels per image was 3.2 for NPA, 120.5 for MA, 0.95 for NV, and 26.5 for Laser.

**Table 2:** Distribution of label frequency and pixel counts in 55 ° and UWF FFA images.

Label name	Frequency counts			Pixel counts		
	55 °	UWF	All	55 °	UWF	All
NPA	1,024	503	1,527	20,946,340	8,731,017	29,677,357
MA	41,741	16,234	57,975	1,519,689	486,512	2,006,201
NV	359	100	459	2,305,044	388,791	2,693,835
Laser	9,509	3,253	12,762	5,659,824	1,257,354	6,917,178

### Primary performance on 55 °FFA images

Model performance metrics, including the Dice, IoU and recall scores for 55 °, UWF FFA, and the overall dataset are presented in Table 3. The detection of NPA, MA, and NV on 55 ° FFA images yielded Dice scores of 0.65 ± 0.24, 0.70 ± 0.13, and 0.73 ± 0.23, respectively. For laser spots, a target not previously emphasized in FFA segmentation studies, the Dice score was 0.70 ± 0.17.

**Table 3:** Model performance metrics on 55 ° FFA, UWF FFA, and the overall dataset.

Label name	55 °			UWF			All		
	Dice	IoU	Recall	Dice	IoU	Recall	Dice	IoU	Recall
NPA	0.65 ± 0.24	0.53 ± 0.24	0.74 ± 0.23	0.48 ± 0.21	0.34 ± 0.17	0.47 ± 0.21	0.64 ± 0.24	0.51 ± 0.24	0.71 ± 0.25
MA	0.70 ± 0.13	0.55 ± 0.14	0.79 ± 0.12	0.58 ± 0.19	0.43 ± 0.17	0.70 ± 0.11	0.69 ± 0.14	0.54 ± 0.14	0.78 ± 0.12
NV	0.73 ± 0.23	0.62 ± 0.26	0.88 ± 0.18	0.50 ± 0.34	0.38 ± 0.30	0.79 ± 0.16	0.72 ± 0.25	0.61 ± 0.26	0.87 ± 0.18
Laser	0.70 ± 0.17	0.56 ± 0.17	0.72 ± 0.20	0.74 ± 0.03	0.59 ± 0.04	0.80 ± 0.09	0.70 ± 0.17	0.56 ± 0.16	0.73 ± 0.20

### Exploratory results on UWF-FFA images

Given the very limited UWF test set (*n* = 10), these results should be interpreted as preliminary exploratory observations rather than robust evidence of cross-modality generalizability. Overlap-based performance for NPA, MA, and NV was lower on UWF than on 55 ° images (Table 3, Supplementary Figure S6), with Dice scores of 0.48 ± 0.21 (*p* = 0.02), 0.58 ± 0.19 (*p* = 0.01), and 0.50 ± 0.34 (*p* = 0.14), respectively. This pattern likely reflects domain shift related to peripheral distortion, image geometry, and limited UWF training exposure. Laser performance remained numerically similar, but this finding should also be interpreted cautiously because of the small sample size.

### Results by disease type

**Figure 2:**
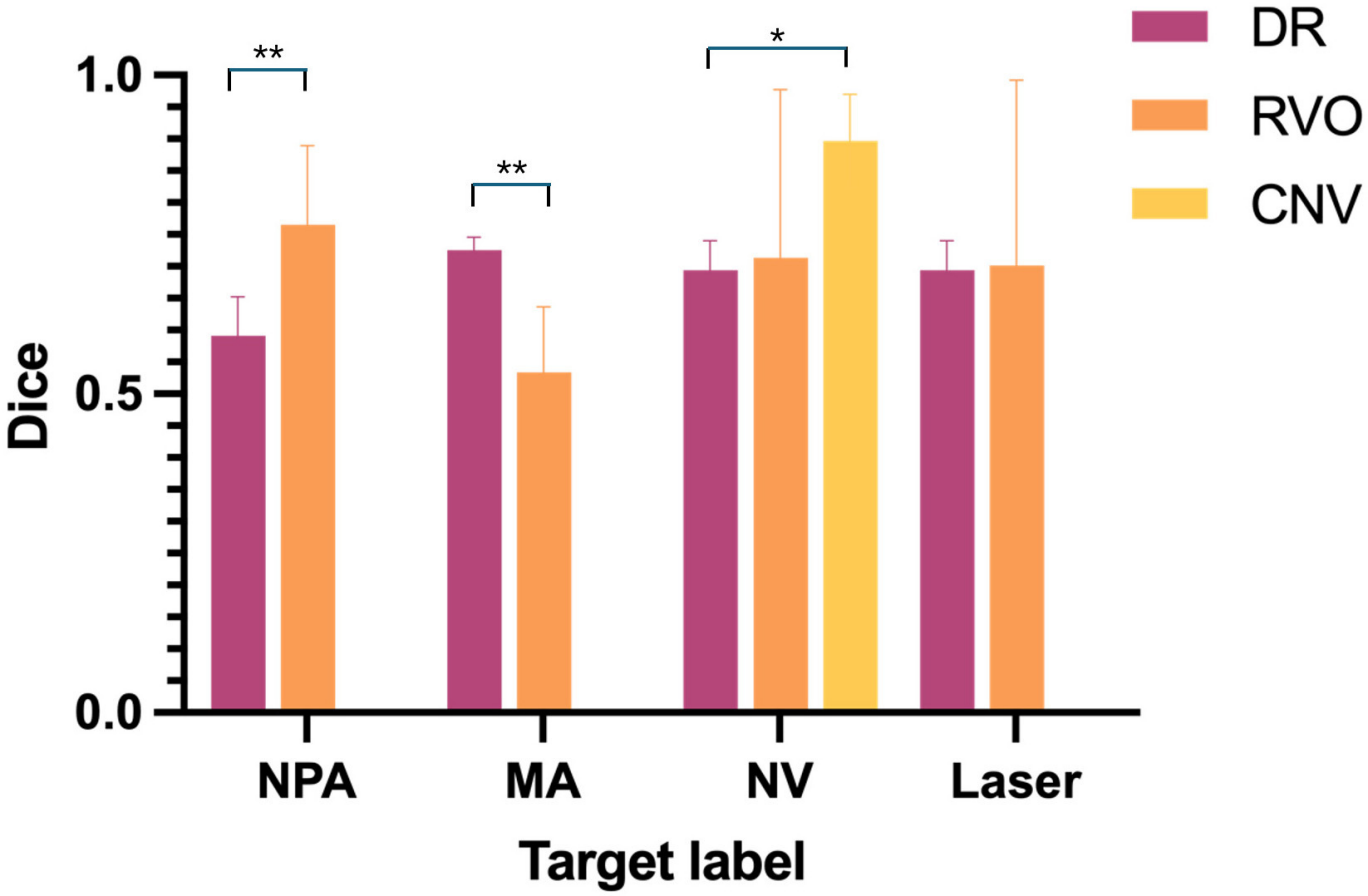
Comparative Dice scores for target labels across DR, RVO, and CNV.

In DR, the Dice scores for NPA, MA, NV, and Laser were 0.59 ± 0.22, 0.73 ± 0.09, 0.66 ± 0.25, and 0.70 ± 0.11, respectively. In RVO, the corresponding Dice scores were 0.77 ± 0.25, 0.53 ± 0.20, 0.71 ± 0.29, and 0.70 ± 0.31. Additionally, in CNV, the Dice score for NV was notably high at 0.90 ± 0.09. Table 4 details performance for each label by disease. Figure 2 highlights the differences in Dice scores, demonstrating a significantly better segmentation performance for NPA in RVO compared to DR, and a superior performance for MA segmentation in DR over RVO (*p* < 0.01). Regarding NV segmentation, performance in CNV was superior to DR and numerically higher than in RVO; only the comparison with DR reached statistical significance (*p* = 0.02).

**Table 4:** Model performance metrics by disease category.

Label name	DR			RVO			CNV		
	Dice	IoU	Recall	Dice	IoU	Recall	Dice	IoU	Recall
NPA	0.59 ± 0.22	0.45 ± 0.20	0.68 ± 0.23	0.77 ± 0.25	0.67 ± 0.28	0.81 ± 0.27	——	——	——
MA	0.73 ± 0.09	0.55 ± 0.10	0.79 ± 0.09	0.53 ± 0.20	0.43 ± 0.17	0.74 ± 0.18	——	——	——
NV	0.66 ± 0.25	0.54 ± 0.26	0.82 ± 0.20	0.71 ± 0.29	0.61 ± 0.29	0.97 ± 0.02	0.90 ± 0.09	0.82 ± 0.14	0.97 ± 0.08
Laser	0.70 ± 0.11	0.54 ± 0.11	0.71 ± 0.15	0.70 ± 0.31	0.60 ± 0.28	0.74 ± 0.33	——	——	——

### Results by phase

**Figure 3:**
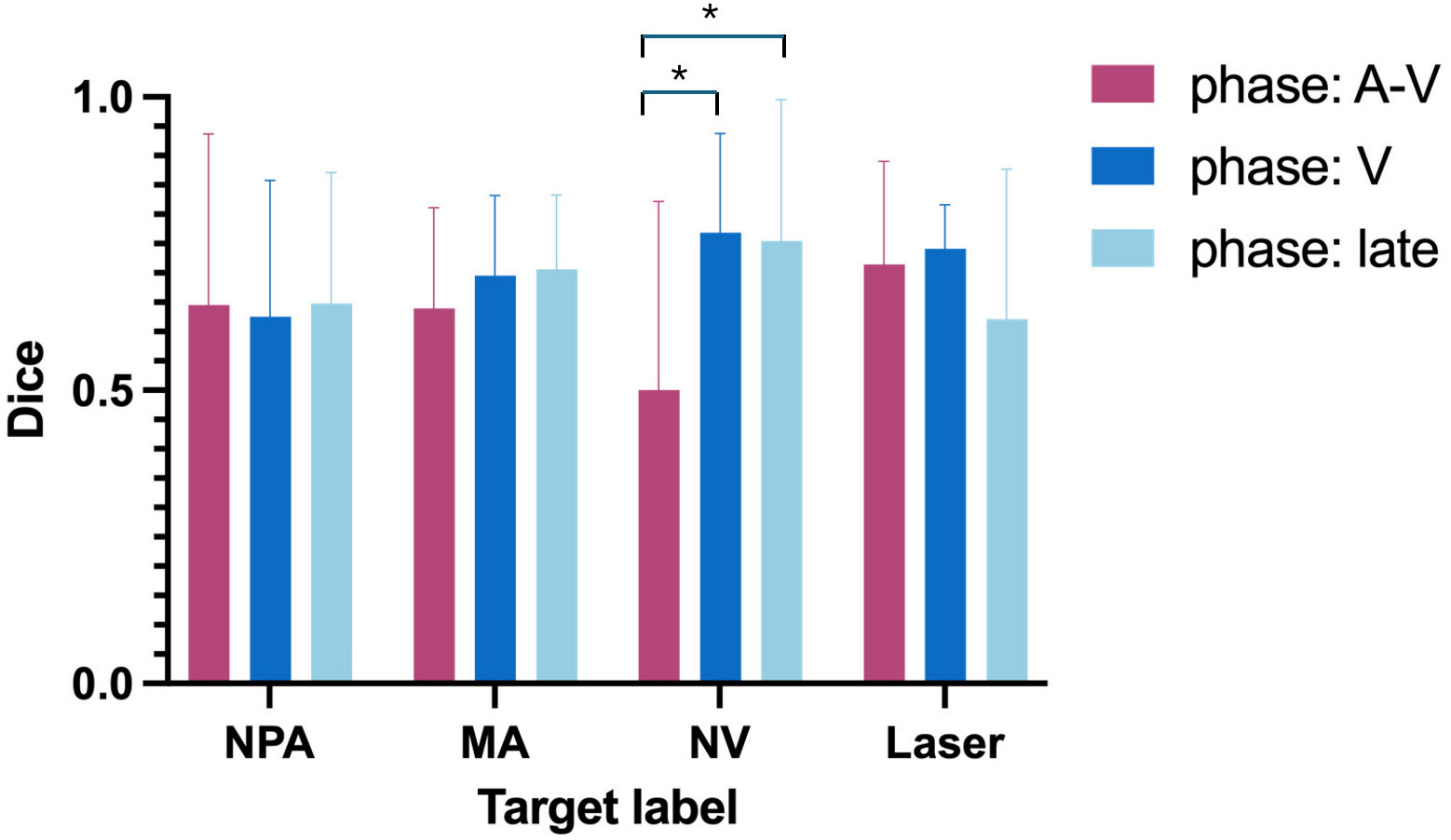
Comparative Dice scores for target labels across arteriovenous, venous, and late phases.

As illustrated in Figure 3, segmentation of NPA, MA, and Laser showed no significant differences across arteriovenous, venous, and late phases. In contrast, NV segmentation was significantly stronger in the venous phase (Dice = 0.77 ± 0.17, *p* = 0.01) and late phase (Dice = 0.75 ± 0.24, *p* = 0.02) than in the arteriovenous phase (Dice = 0.50 ± 0.32). Detailed model performance metrics by phase are provided in Table 5.

**Table 5:** Model performance metrics by phase.

Label	A-V			V			late		
name	Dice	IoU	Recall	Dice	IoU	Recall	Dice	IoU	Recall
NPA	0.65 ± 0.29	0.53 ± 0.29	0.77 ± 0.22	0.63 ± 0.23	0.49 ± 0.23	0.68 ± 0.25	0.65 ± 0.22	0.51 ± 0.23	0.72 ± 0.26
MA	0.64 ± 0.17	0.49 ± 0.16	0.72 ± 0.16	0.70 ± 0.14	0.55 ± 0.14	0.78 ± 0.11	0.71 ± 0.13	0.56 ± 0.14	0.82 ± 0.10
NV	0.50 ± 0.32	0.39 ± 0.32	0.78 ± 0.26	0.77 ± 0.17	0.65 ± 0.21	0.86 ± 0.18	0.75 ± 0.24	0.65 ± 0.26	0.92 ± 0.11
Laser	0.71 ± 0.18	0.58 ± 0.19	0.75 ± 0.16	0.74 ± 0.08	0.59 ± 0.09	0.76 ± 0.15	0.62 ± 0.26	0.49 ± 0.22	0.64 ± 0.28

### Qualitative evaluation

Representative examples of model predictions and ground-truth masks for different labels in 55 ° FFA images are shown in Supplementary Figures S1–S5. Overall, the segmentation performance was satisfactory across lesion types. NPA recognition is particularly strong, especially in BRVO cases. MA predictions generally aligned with ground truth, although false positives occurred in some cases, such as mistaking parts of laser spots with window defects for MA or interpreting punctate hyperfluorescence from BRVO depigmentation as MA. The model accurately identified typical NV, but performance decreased in early-stage lesions or when leakage was subtle, occasionally confusing prominent vascular leakage with NV. Laser spot segmentation was largely satisfactory; however, lower Dice scores were mainly observed in atypical cases, and some predictions correctly identified laser spots that had been missed in the original ground truth. In UWF examples (Supplementary Figure S5), NPA detection was less accurate in the peripheral retina, and MA/NV performance was inferior to that on 55 ° images, further supporting the exploratory nature of the UWF analysis.

### Ablation study: impact of training set size on model performance

**Figure 4:**
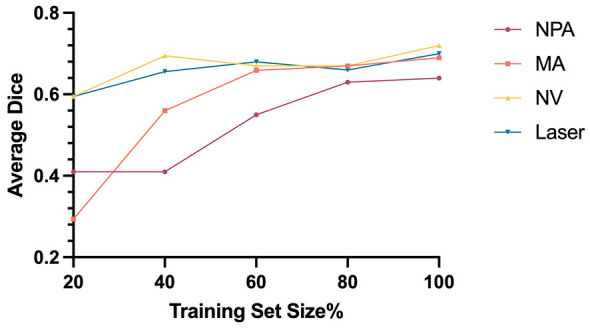
Influence of training-set size on average Dice scores for lesion segmentation.

To evaluate the impact of training-set size, we further divided the original training set into subsets, containing 20%, 40%, 60%, and 80% of the full dataset. As depicted in Figure 4, average model performance for the labels NPA, MA, NV, and Laser consistently improved as the amount of the training data increased.

## Discussion

In this work, we developed a unified deep learning framework for simultaneous segmentation of NPA, MA, NV, and laser spots across multiple retinal vascular diseases. Rather than proposing a novel backbone architecture, the key novelty of this study lies in the clinical task formulation: a single framework that addresses multiple morphologically distinct lesions across DR, RVO, and CNV. To our knowledge, this is also the first study to explicitly segment post-photocoagulation laser spots as a dedicated class, which is clinically important for reducing false positives and estimating the true residual ischemic burden in treated eyes. On standard 55 ° images, the model achieved overall satisfactory performance and approached the range of human inter-grader agreement.

The UWF findings should be interpreted conservatively. Because the exploratory UWF cohort included only 53 images, with 10 test images, it is statistically insufficient to support strong claims of cross-modality generalizability. We therefore repositioned UWF as a preliminary exploratory analysis rather than as a major validation arm. The lower UWF Dice scores, particularly for NPA, most likely reflect a combination of peripheral distortion, resolution and aspect-ratio differences, and limited modality-specific training data. These findings are better understood as evidence of domain shift and as justification for future UWF-specific or multi-center expansion, rather than as proof of deployable UWF performance.

When comparing our results with highly specialized prior studies, our overall 55 ° NPA Dice score of 0.65 is lower than the best disease-specific binary models. This difference is likely explained by three factors. First, Tang et al. focused exclusively on RVO, in which NPA often has clearer and more continuous boundaries than the patchy, diffuse NPA frequently seen in DR (18–20, 22, 24). Second, our dataset was intentionally not over-curated and retained clinically realistic obscuration such as hemorrhagic masking. Third, our framework jointly optimizes multiple lesion classes, which introduces an inherent performance trade-off compared with single-task models. It is worth noting that we grouped retinal and choroidal neovascularization under a single ‘NV' category. Despite biological differences, they share key angiographic features—hyperfluorescence and leakage—allowing the model to successfully extract shared visual representations. Regarding phase-dependent performance, the superior NV performance in venous and late phases is consistent with our annotation protocol, because the NV label included associated fluorescein leakage rather than only the vascular scaffold itself. Regarding NPA quantification, we acknowledge that the arteriovenous phase remains the clinical gold standard due to optimal contrast, and late-phase measurements were included primarily to test model stability against leakage artifacts. Our ablation study confirms that larger training sets correlate with higher performance, a trend supported by Zhao et al., who achieved superior NPA segmentation with the largest dataset of 1,295 images (15).

Establishing a definitive ground truth for FFA segmentation is inherently challenging due to subjective interpretation. Inter-grader variability is a well-documented issue; for instance, Joan et al. reported a Dice score of 0.65 for NPA between experts, and Lee et al. noted similarly variable IoUs (18, 24). Consistent with these findings, our internal assessment revealed an inter-grader Dice score of 0.69. This creates a ceiling effect for supervised learning: when experts do not completely agree on the precise boundary of diffuse or microscopic lesions, the model cannot be expected to consistently exceed the consensus it is trained on. In this context, the model's performance suggests that it is approximating expert consensus rather than learning an absolute biological truth. Therefore, rather than creating a new ground truth, the model offers value by providing a standardized, reproducible baseline. This capability is crucial for mitigating the inconsistency inherent in manual grading and reducing the labor burden.

Clinically, a consistent segmentation system could reduce manual grading burden, support less experienced readers, and facilitate more standardized quantification of ischemic and neovascular lesions. Quantification of NPA is particularly relevant for risk stratification and treatment planning. In CRVO, eyes with NPAs exceeding 10-disc areas on 55 ° FFA images are at a higher risk of developing anterior segment NV (14), while an initial NPA over 75-disc areas on UWF FFA images greatly increases the chances of progressing to ischemic CRVO within the 1 year (28, 29). By enabling more reproducible lesion quantification, automated segmentation may help identify eyes that warrant closer surveillance or earlier intervention.

Our study has several limitations. First, the strongest evidence in this manuscript comes from the 55 ° dataset; the UWF cohort was too small for robust validation and is reported only as an exploratory observation. Second, the lack of external validation may limit generalizability. Third, incorporating paired color fundus images could better differentiate hemorrhage from NPA. Future work should expand the dataset across centers and camera platforms and may benefit from semi-supervised learning, active learning, and federated learning to reduce annotation burden while improving robustness.

In conclusion, our study demonstrates the feasibility of a deep learning-based framework for multi**-**lesion segmentation in FFA images across a variety of retinal vascular diseases, with the most robust evidence derived from standard 55 ° images. The model exhibits overall satisfactory performance for NPA, MA, NV, and laser spots and provides a highly consistent and reproducible approach to lesion quantification. With broader external validation and larger multi-center datasets, this framework may support more standardized FFA interpretation and improve risk stratification in retinal vascular disease.

## Data Availability

The original contributions presented in the study are included in the article/Supplementary material, further inquiries can be directed to the corresponding authors.
