# Supplementary material for: Automated interpretation of fundus fluorescein angiography with multi-retinal vascular lesion segmentation
Source: Front Med (Lausanne). 2026 Apr 7;13:1762735. doi: 10.3389/fmed.2026.1762735 (PMC13095574; doi:10.3389/fmed.2026.1762735)

Figure S1. Visualization of NPA predictions versus ground truth in 55° FFA images. FFA, fundus fluorescein angiography; NPA, non-perfusion areas; GT, ground truth.


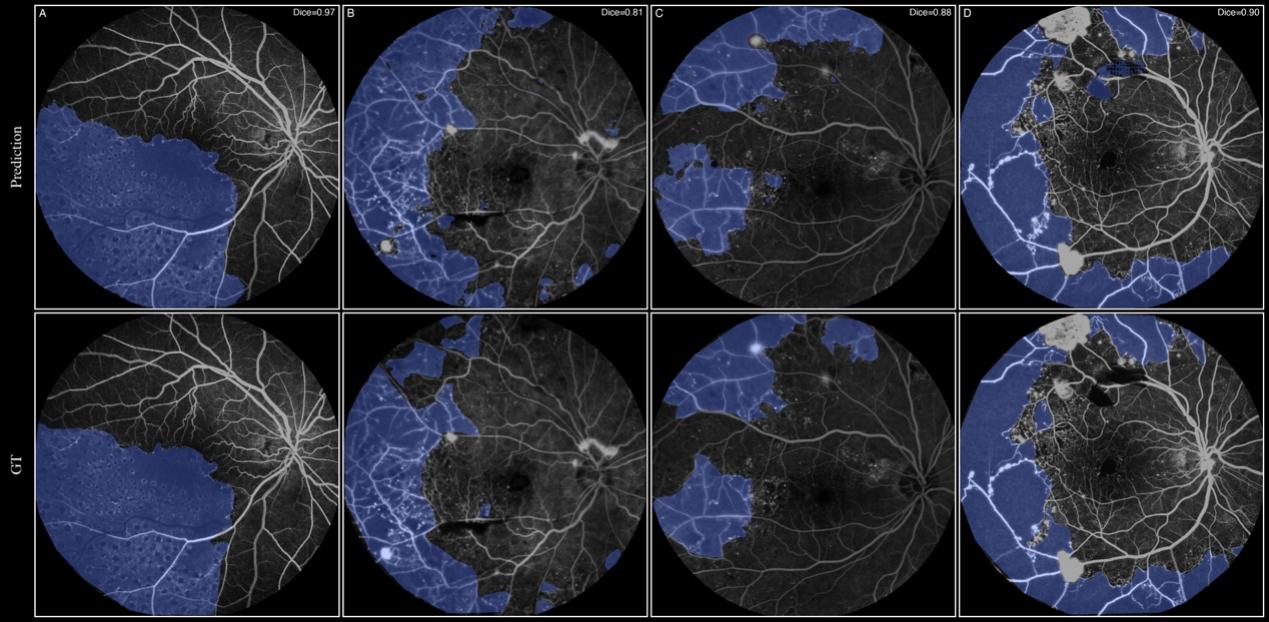


Figure S2. Visualization of MA predictions versus ground truth in 55° FFA images. FFA, fundus fluorescein angiography; MA, microaneurysms; GT, ground truth.


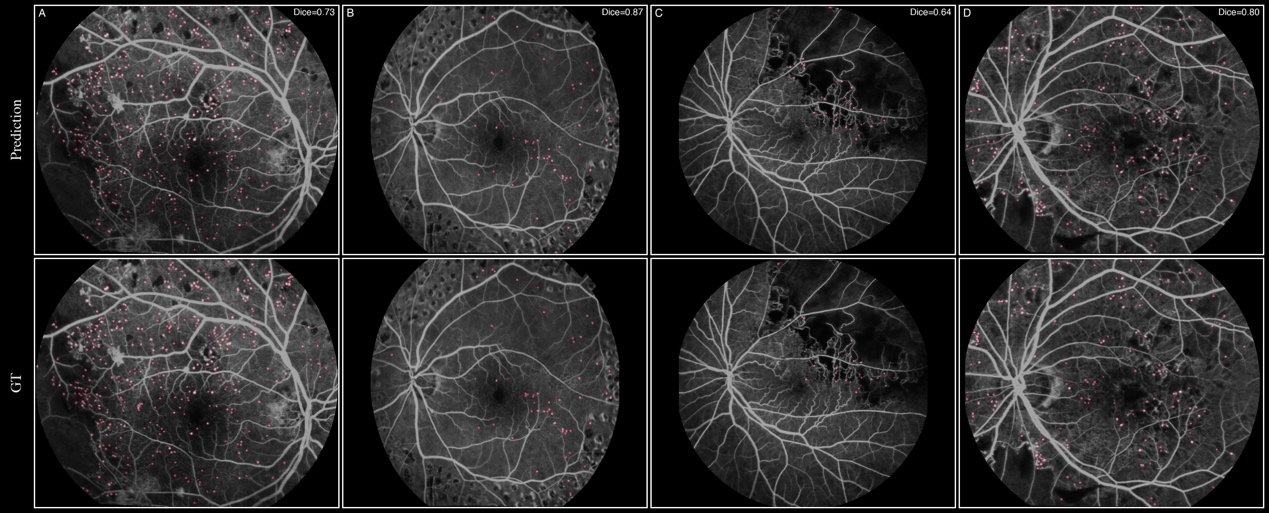


Figure S3. Visualization of NV predictions versus ground truth in 55° FFA images

FFA, fundus fluorescein angiography; NV, neovascularization; GT, ground truth.


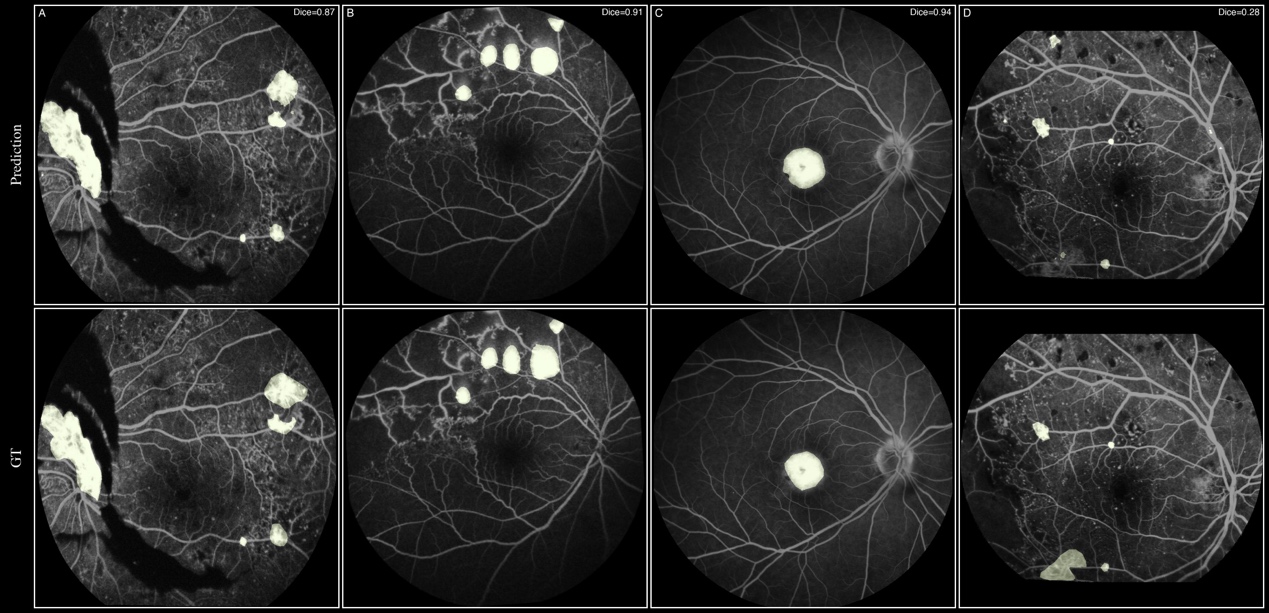


Figure S4. Visualization of predicted laser spot versus ground truth in 55° FFA images

FFA, fundus fluorescein angiography; GT, ground truth.


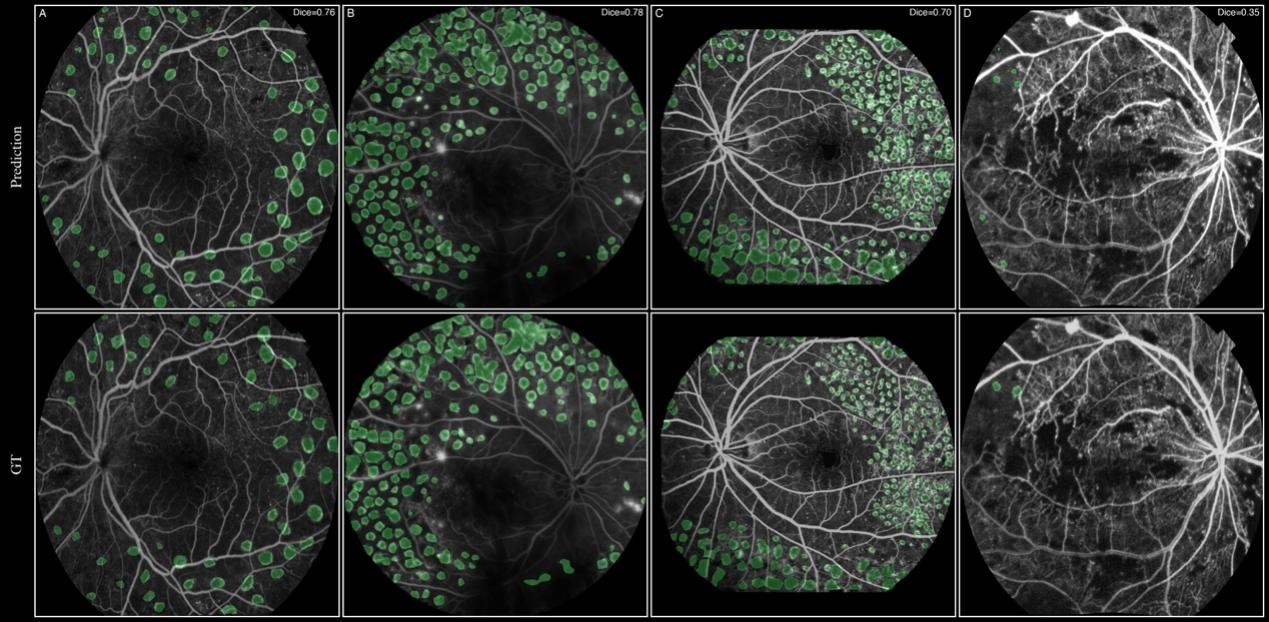


Figure S5. Visualization of target label predictions versus ground truth in UWF FFA images. (A) NPA. (B)MA. (C) NV (D) Laser spots. UWF, ultra-widefield imaging; FFA, fundus fluorescein angiography; NPA, non-perfusion areas; MA, microaneurysms; NV, neovascularization; GT, ground truth.


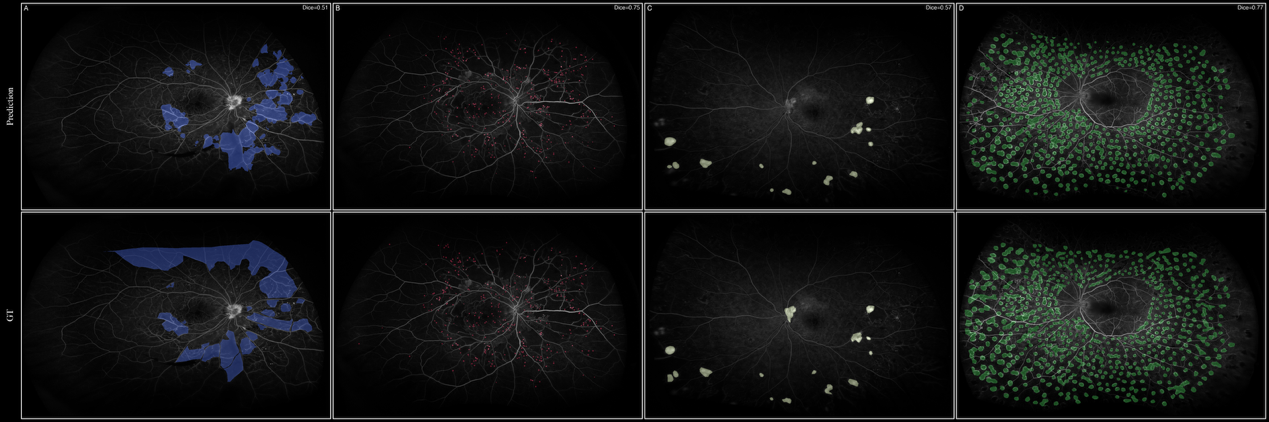


Figure S6. Grouped comparison of Dice scores between 55°and UWF FFA images across lesion types.
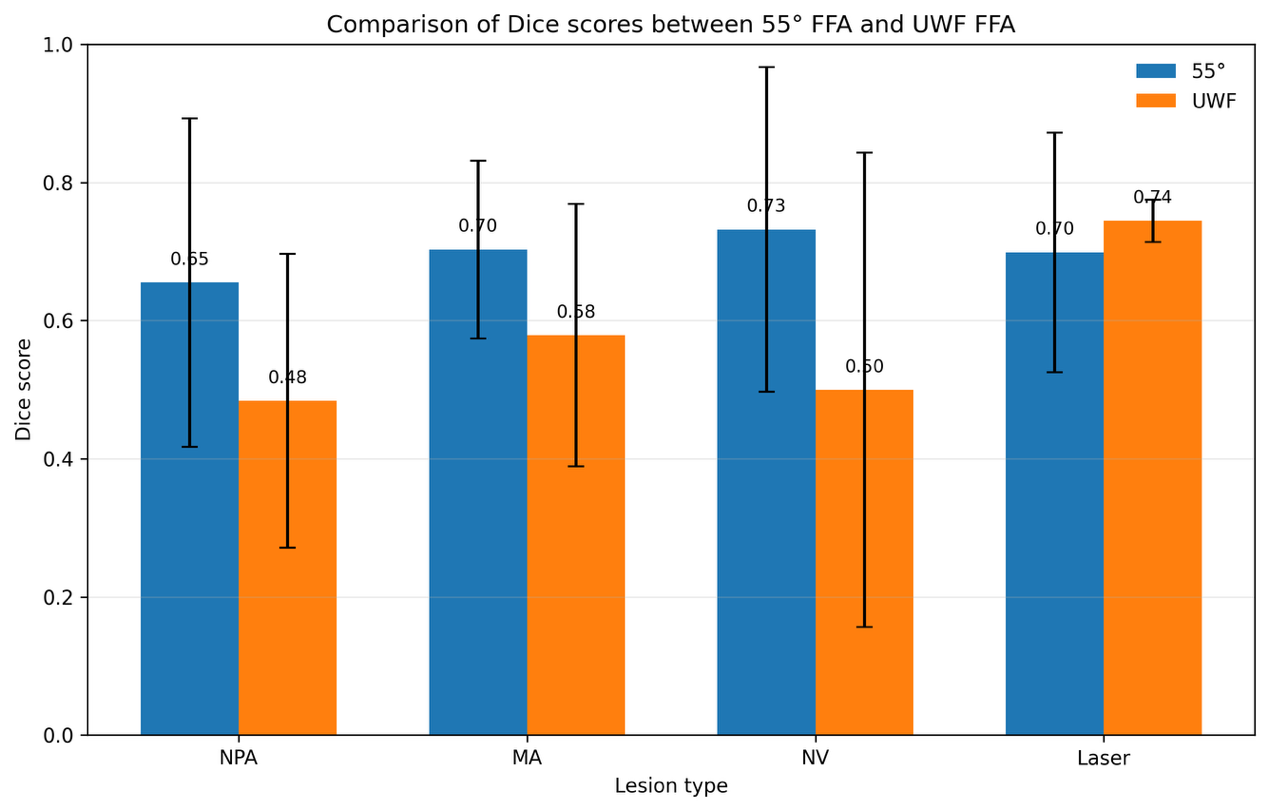

Supplement: Supplementary file 1 [file Table_1.docx]
