# Supplementary material for: Automated interpretation of fundus fluorescein angiography with multi-retinal vascular lesion segmentation
Source: Front Med (Lausanne). 2026 Apr 7;13:1762735. doi: 10.3389/fmed.2026.1762735 (PMC13095574; doi:10.3389/fmed.2026.1762735)
Supplement: Supplementary file 2 [file Table_2.docx]

Table 1: Summary of previous studies on lesion segmentation performance in FFA images.

| **Year** | **Author** | **Modality** | **Target** | **Disease** | **Test Model** | **Best F1 score** |
| --- | --- | --- | --- | --- | --- | --- |
| 2020 | Joan  et al. | UWF | NPA | DR | U-net | 0.661 |
| 2020 | Jin  et al. | 30° | NPA | DME | U-net | N/A |
| 2021 | Tang  et al. | 55° | NPA | RVO | CE-net,  U-Net, DeepLabv3+ | 0.883 |
| 2021 | Chen  et al. | 55° | Leakage | CSC | attention gated network | 0.811 |
| 2022 | Kanato  et al. | 55° | NPA | RVO | U-Net, PSPNet, DeepLab v3+ | 0.736 |
| 2022 | Xiang  et al. | 55° | NPA | Not Specified | ResNet50+VGG16+MLFB+SAT | 0.772 |
| 2022 | Lee  et al. | UWF | NPA  NV | DR | DeepLab v3+, SegNet,  U-net | 0.67 0.87 |
| 2023 | David  et al. | 55° | NV  Leakage | nAMD | U-net based | 0.73  0.65 |
| 2023 | Zhao  et al. | 55° | NPA | DR&  BRVO | ResNet-152, Unet-VGG16 | 0.90 |

FFA, fundus fluorescein angiography; UWF, ultra-widefield imaging; DR, diabetic retinopathy; RVO, retinal vein occlusion; BRVO, branch retinal vein occlusion; CSC, central serous chorioretinopathy; nAMD, neovascular age-related macular degeneration; NPA, non-perfusion areas; NV, neovascularization;

Table 2. Distribution of label frequency and pixel counts in 55° and UWF FFA images.

| Label name | Frequency counts | | | Pixel counts | | |
| --- | --- | --- | --- | --- | --- | --- |
|  | 55° | UWF | All | 55° | UWF | All |
| NPA | 1024 | 503 | 1527 | 20946340 | 8731017 | 29677357 |
| MA | 41741 | 16234 | 57975 | 1519689 | 486512 | 2006201 |
| NV | 359 | 100 | 459 | 2305044 | 388791 | 2693835 |
| Laser | 9509 | 3253 | 12762 | 5659824 | 1257354 | 6917178 |

UWF, ultra-widefield imaging ; NPA, non-perfusion areas; MA, microaneurysms; NV, neovascularization; Laser, laser spots;

Table 3. Model performance metrics on 55° FFA, UWF FFA, and the overall dataset.

| **Label name** | **55°** | | | **UWF** | | | **All** | | |
| --- | --- | --- | --- | --- | --- | --- | --- | --- | --- |
|  | **Dice** | **IoU** | **Recall** | **Dice** | **IoU** | **Recall** | **Dice** | **IoU** | **Recall** |
| NPA | 0.65±0.24 | 0.53±0.24 | 0.74±0.23 | 0.48±0.21 | 0.34±0.17 | 0.47±0.21 | 0.64±0.24 | 0.51±0.24 | 0.71±0.25 |
| MA | 0.70±0.13 | 0.55±0.14 | 0.79±0.12 | 0.58±0.19 | 0.43±0.17 | 0.70±0.11 | 0.69±0.14 | 0.54±0.14 | 0.78±0.12 |
| NV | 0.73±0.23 | 0.62±0.26 | 0.88±0.18 | 0.50±0.34 | 0.38±0.30 | 0.79±0.16 | 0.72±0.25 | 0.61±0.26 | 0.87±0.18 |
| Laser | 0.70±0.17 | 0.56±0.17 | 0.72±0.20 | 0.74±0.03 | 0.59±0.04 | 0.80±0.09 | 0.70±0.17 | 0.56±0.16 | 0.73±0.20 |

UWF, ultra-widefield imaging ; NPA, non-perfusion areas; MA, microaneurysms; NV, neovascularization; Laser, laser spots; Dice, Dice scores; IoU, Intersection over Union;

Table 4. Model performance metrics by disease category.

| **Label name** | **DR** | | | **RVO** | | | **CNV** | | |
| --- | --- | --- | --- | --- | --- | --- | --- | --- | --- |
|  | **Dice** | **IoU** | **Recall** | **Dice** | **IoU** | **Recall** | **Dice** | **IoU** | **Recall** |
| NPA | 0.59±0.22 | 0.45±0.20 | 0.68±0.23 | 0.77±0.25 | 0.67±0.28 | 0.81±0.27 | —— | —— | —— |
| MA | 0.73±0.09 | 0.55±0.10 | 0.79±0.09 | 0.53±0.20 | 0.43±0.17 | 0.74±0.18 | —— | —— | —— |
| NV | 0.66±0.25 | 0.54±0.26 | 0.82±0.20 | 0.71±0.29 | 0.61±0.29 | 0.97±0.02 | 0.90±0.09 | 0.82±0.14 | 0.97±0.08 |
| Laser | 0.70±0.11 | 0.54±0.11 | 0.71±0.15 | 0.70±0.31 | 0.60±0.28 | 0.74±0.33 | —— | —— | —— |

DR, diabetic retinopathy; RVO, retinal vein occlusion; CNV, choroidal neovascularization; NPA, non-perfusion areas; NV, neovascularization; MA, microaneurysms; Laser, laser spots; Dice, Dice scores; IoU, Intersection over Union;

Table 5. Model performance metrics by phase.

| **Label** | **A-V** | | | **V** | | | **late** | | |
| --- | --- | --- | --- | --- | --- | --- | --- | --- | --- |
| **name** | **Dice** | **IoU** | **Recall** | **Dice** | **IoU** | **Recall** | **Dice** | **IoU** | **Recall** |
| NPA | 0.65±0.29 | 0.53±0.29 | 0.77±0.22 | 0.63±0.23 | 0.49±0.23 | 0.68±0.25 | 0.65±0.22 | 0.51±0.23 | 0.72±0.26 |
| MA | 0.64±0.17 | 0.49±0.16 | 0.72±0.16 | 0.70±0.14 | 0.55±0.14 | 0.78±0.11 | 0.71±0.13 | 0.56±0.14 | 0.82±0.10 |
| NV | 0.50±0.32 | 0.39±0.32 | 0.78±0.26 | 0.77±0.17 | 0.65±0.21 | 0.86±0.18 | 0.75±0.24 | 0.65±0.26 | 0.92±0.11 |
| Laser | 0.71±0.18 | 0.58±0.19 | 0.75±0.16 | 0.74±0.08 | 0.59±0.09 | 0.76±0.15 | 0.62±0.26 | 0.49±0.22 | 0.64±0.28 |

Phase:A-V, arteriovenous phase; Phase:V, venous phase; NPA, non-perfusion areas; MA, microaneurysms; NV, neovascularization; Laser, laser spots; Dice, Dice scores; IoU, Intersection over Union;
